# Compassion focused therapy for older people: Why it is needed and adaptations for clinical practice

**DOI:** 10.1111/papt.12579

**Published:** 2025-03-10

**Authors:** Rebecca Poz, Catriona Craig

**Affiliations:** ^1^ West Suffolk Older Adult Services Norfolk & Suffolk NHS Foundation Trust Bury St. Edmunds UK; ^2^ Department of Clinical Psychology, Norwich Medical School University of East Anglia Norwich UK; ^3^ Buckinghamshire Older Adult Psychology Service Oxford Health NHS Foundation Trust High Wycombe UK

**Keywords:** anxiety, CFT, compassion‐focused therapy, complex emotional needs, dementia, depression, older people, trauma

## Abstract

**Background:**

Compassion‐focused therapy (CFT) has an emerging evidence base and is becoming an increasingly popular therapeutic modality. The journey through later life poses individuals with various challenges to navigate, including loss of roles and relationships, deteriorating physical health and cognition and death of friends and family members. In addition to any unprocessed challenges lived through in earlier life. Later life is also a unique period where reflection on one's life experiences and choices can occur, which can lead to feelings of regret, disappointment and shame for some, whilst simultaneously facing ageism and barriers to accessing therapy. CFT is well‐placed to facilitate older people to face these challenges by exploring their relationship to themselves and others as they navigate ageing. This is increasingly important as we are living longer and more and more older people develop conditions where they will require care. Developing greater compassion for oneself and allowing ourselves to be cared for by others may facilitate a smoother journey and minimise distress.

**Methodology:**

The paper summarises the published work trialling CFT across a range of older patient groups, which shows that older people are open to a CFT approach, they find it an acceptable intervention and it has had wide‐reaching benefits. There remains a paucity of high‐quality research delivering CFT to older people which limits our conclusions of its effectiveness.

**Recommendations:**

Recommendations of ways in which CFT can be adapted for older people and those living with dementia, consistent with Gilbert's therapeutic themes (2022), are provided.

## INTRODUCTION

Compassion Focused Therapy (CFT) is a psychotherapy which aims to reduce clinical levels of distress in people through cultivating instinctive ways of responding to threatening situations. CFT is a biopsychosocial approach which is informed by evolution and social mentality theory. Unlike other primates full‐term human babies are born relatively helpless and without significant defence mechanisms (Mitteroecker & Fischer, 2024). This required the evolution of neurobiological mechanisms to maintain the proximity between the mother and infant, typically described as attachment, which underpins the co‐evolution of care‐giving and care‐seeking as a social mentality. The formation of attachment begins in the womb and extends across the lifespan. However, the presence of an attachment figure is insufficient, rather the quality of early parent attachment has been shown to influence brain circuitry and plasticity, as well as subsequent capacities for emotion regulation throughout life (Perry et al., 2017).

Over the course of the last 30 years, Gilbert has developed CFT building on social mentality theory and the associated evolved abilities (Gilbert, 1995, 2000, 2020); to identify our own distress, to turn to others at times of distress, to be able to read signals of distress in others and to have the evolved competences to comfort and provide safeness to others and over the course of childhood development to provide this soothing to ourselves. CFT has now been demonstrated, including through a series of meta‐analyses (Petrocchi et al., 2024), to have clinical efficacy across very many mental health conditions (i.e. Craig et al., 2020; Millard et al., 2023), and there are few areas in which CFT has not been researched. It has also been shown to be helpful for non‐clinical needs, such as for health care staff (Beaumont et al., 2016), within school settings (Maratos et al., 2019) and Pedagogy (Harvey et al., 2020).

### The invisibility of older people

Although this paper focuses on CFT with older adults – which is an individualised therapy – the two psychologies of CFT are valuable for both ‘noticing’ and ‘doing something about’ the barriers that older people face in even reaching the therapy room.

From the outset it has to be acknowledged that there is a paucity of published papers focused on the delivery of CFT with older people. This is a reflection of many factors. Our society carries with it a dysconscious ageism which infiltrates all aspects, from referral rates of older people into mental health services to a skew in research populations. Whilst the authors acknowledge their UK‐centric bias, contrary to conventional wisdom, ageism towards older people is no less negative in the East (North & Fiske, 2015). Ageism is nuanced across personal beliefs as well as cultural attitudes (Vauclair et al., 2017), and a recent report the WHO identified that one in two people globally hold moderately or highly ageist attitudes (WHO, 2021).

Older adults are at at least the same risk of mental health difficulties as adults of working age, and those older people who live in nursing or residential care are at an increased risk of poor mental health (Age Cymru, 2023). Yet older adults are less likely to seek professional help due to a range of factors including internalised stigma and negative beliefs about mental health (Elshaikh et al., 2023), combined with a sense of ‘hopelessness’ on the part of those who would refer them for treatment and prioritising the physical health of the older people over their mental health (Frost et al., 2019), within a functionalist society which assigns greater importance to those it deems ‘serve a role’ (working age adults) or those due to ‘fulfil a role’ (children and young people) (Powell, 2024) results in lower referral rates for therapy. This flies in the face of the facts that older people are more likely to complete treatment (74% compared to 68% of the general population) and achieve higher recovery rates than the working‐age population (60% compared to 46%) (Burns, 2017). Yet approximately 70% of older adults with mental health conditions do not receive treatment (Brenes et al., 2015).

It has been shown that older adults carry 60% of the national disease burden but represent only 32% of patients in phase II and III clinical trials (Herrera et al., 2011). Recent reviews have demonstrated that 92% of Randomised Control Trials (RCTs) either explicitly excluded older adults or had criteria indirectly limiting their enrolment (Kłosowska et al., 2024). Other researchers have identified a range of barriers to older adults participating in mental health clinical trials; including institutional barriers, study design barriers, collaboration‐related barriers, practical barriers and patient barriers (Newmark et al., 2020).

Ageism also pervades the profession of Clinical Psychology (Bryant & Koder, 2015), which is a key provider of psychological therapies, including CFT, within the National Health Service (NHS). Despite the fact that in the UK nearly a fifth (19.5%) of the population is aged 65 years or older, and although they are consumers of 50% of health and social care spending, it is estimated that around 5% of psychological professionals work with older people across the UK (BPS, 2006). This has not been audited since 2006 (Dow, 2024) although the comparison of memberships of the BPS Division of Clinical Psychology (c. 10,000) and the Faculty of Psychology for Older People standing (c. 550) goes some way to showing the discrepancy. And sadly shows almost no change over the last two decades. Trying to understand drivers at the start of Clinical Psychologists' careers, Lee et al. (2003) identified that many trainees believe that clinical psychology, despite many recent advances, has less to offer older people than other age groups. The responses contain evidence of both ageism and their own fear of ageing and death (Lee et al., 2003). This pattern is mirrored in the American Psychological Association, where only 1.2%–4% of those surveyed described geropsychology as their specialty area, (Hoge et al., 2016; Moye et al., 2019). Similar figures were obtained from an Australian survey, in which 6% of psychologists surveyed indicated that they were specialists in aged care, with 40% of the sample indicating that they had no contact with older clients (Koder & Helmes, 2008).

This introduction aims to set the scene for the paucity of published research into CFT with older people, whilst moving into the rationale for why we should anticipate the value of CFT for older recipients of therapy, and also for the delivery of the therapy.

### The relevance of CFT to the system

As noted, there is a significant gap between the clinical need, the treatment preferences expressed by older people and the response from the wider system. We are living in a global community where one in six older people worldwide are experiencing abuse, often perpetrated by their own carers (WHO, 2023; Yon et al., 2017), and a quarter of older people are socially isolated and lonely. As fellow members of society, we have optional roles in tolerating this, turning a blind eye or striving for change.

In CFT, in order to effect change, we are always thinking about creating and cultivating the conditions that are required to make flourishing possible. With an understanding that any Fears, Blocks and Resistances (FBRs) to giving or receiving compassion are not our fault, but they are our responsibility to work through. Given the identified commonplace avoidance of working with older people, it is important to understand the culturally prevalent FBRs of extending compassion towards our elders, if we are to create a workforce who want to and are skilled in working with older people. Although not therapeutically spelcific to CFT, one of the key engagement competencies developed during CFT is ‘distress tolerance’; which enables us not to avoid, deny or distort painful realities but to ‘stay with our sympathetic reactions’ (Gilbert, 2022a, 2022b).

The main predictors of ageism have been reported to be a high level of anxiety related to ageing, a low level of knowledge about ageing and a limited number of contacts with the elderly; where quality is more important than quantity (Podhorecka et al., 2022). Public health messaging promoting awareness of ageing in helpful and factual ways could be an appropriate systemic response to support distress tolerance towards later life.

### The value of CFT to the therapist

The noted lack of desire to work with older people will hold relevance to whichever therapeutic modality is being practiced by the clinician, not just those offering CFT. But where it holds particular relevance to CFT, is that within CFT we consider three ‘flows’ of compassion; compassion towards others, compassion from others and compassion towards the self (Gilbert, 2022a, 2022b). This connection between concept of self and concept of other, and being able to hold both with compassion, has been quantified by Podhorecka et al. (2022), where the individual young adult's feelings towards themselves, as measured using a self‐esteem scale, was positively correlated with their perception of elderly others and their perception of older age.

Within the world of CFT, compassion is defined as having two strands; that of noticing, being alert to and aware of suffering both in oneself and in others; being ‘engagement focused’ and a second strand of embracing the courage to turn towards the suffering and do something to alleviate it; being ‘action focused’ (Gilbert, 2022a, 2022b). Although all therapy in clinical practice works with different forms of suffering, the therapist working with older people will routinely need to turn towards the suffering of senescence and multiple losses, as well as tolerating their own mortality. CFT provides a supportive framework in which this is possible.

### The relevance of CFT for older people

#### Three circles becoming unbalanced

One of the ways of formulating and making sense of the person's experience, within CFT is using the ‘three circles model’; which conceptualises how the system is currently regulating the person's emotions based on evolved motivations. One of the evolved motivations of humans is to survive by identifying potential threats to safety, another is to survive through identifying sources of beneficial resources and a third is to identify sources of social safeness. The ‘aim’ is of these three regulation systems is to be responsive moment‐by‐moment to context, such that the threat system becomes helpfully dominant in response to an external threat, and the drive system dominates in response to external competition. However, once the external trigger passes the three systems should come into balance with each other, akin to a three‐legged stool. Following this analogy compassionate wisdom is the seat with oversight of the three systems, as well as of the prevailing environment to make predictions about which system requires more focus in order to respond in the most compassionate way to the present moment and maintain attunement.

Ageing itself may affect each of the three affect‐regulation systems, through repeated over‐demand on the same system or reduced opportunity to activate a system.

- Threat‐Focused: the world around an older person may literally become more threatening; they are more likely to be victims of fraud (Re‐engage, 2023), more likely to be recipients of abuse (WHO, 2024) especially women (NIA, 2023), sexual violence occurs towards 1%–2% of older men and women (Nobels et al., 2020), the environment may pose more threats in the form of trip hazards and extreme temperatures, and the body itself may become ‘threatening’ as physical health declines. And these factors interact; for example, the oldest old and those with worse mental, cognitive and physical health are the most susceptible to fraud (Solway et al., 2023), abuse (NIA, 2023) and the environment (GOV.UK, 2022). Loneliness, being isolated and dependent has been identified by ‘experts by experience’ as creating the risks of their abuse (Mysyuk et al., 2016). For those with dementia, the diagnosis itself can pose a threat to the person's identity (Clare, 2011), and the changes in cognition that make it so much harder to navigate the world can lead to feelings of embarrassment, shame, self‐criticism and a fear of social exclusion (Cheston, 2005).
- Resource‐focused: opportunities to access resources and a range of resources may become fewer. There may be a narrowing of opportunities, or a removal of social permission to experience activities triggering hedonic emotions, such as sexual activity or eudemonic emotions, such as challenging projects. Retirement is usually resulting in less financial stability and fewer opportunities to engage in the same cognitive and social challenges. Access to non‐employment based activities may be limited by finances, possible changes in the ability to access a vehicle and the local limitations of public transport.
- Affiliative‐focused: key relationships and sources of interpersonal safeness may be taken away through bereavement, retirement or the inability to travel and even opportunities to have informal social interactions such as waving at neighbours or chatting in a checkout queue may reduce or vanish. Opportunities to care for others may become extreme; either vanishing or being incessant and opportunities for playfulness and compassionate touch, shown to increase heart rate variability through C‐tactile fibre stimulation (Triscoli et al., 2017), may dwindle to extinction.

In addition to the ageing process itself affecting the re‐balancing of the emotion regulation systems, there may also be cohort vulnerabilities that can leave older people at a greater risk of relating to themselves and others in threat‐based ways. Our current cohort of older people grew up in the 1940s, 50s and 60s, at which time authoritarian and strict parenting styles and child‐rearing approaches were more common (i.e. Trifan et al., 2014). This may have taken the form of a lack of emotional‐responsiveness, dismissing the emotional needs of the child and expecting obedience. This lack of closeness and a sense of emotional and physical safety with the parent/s may have led those children and later adults not only to not understand their own emotions but also to cut them off and to not expect that others are a source of safety or comfort, or may even be a threat. Typically, this can lead to an over‐developed sense of threat and an under‐developed ability to soothe oneself or to accept care from others. Approximately 6.5% of older people had early trauma histories of sexual abuse (Draper et al., 2008), which historically were swept under the carpet and ‘forgotten about’ as so not to bring (perceived) shame on families. Despite the positive shift in speaking up about childhood abuse in the last decade, these early shame‐based memories of the abuse itself and loved ones' dismissive responses have had little or no opportunities to be processed or de‐shamed resulting in lifelong impacts on physical health and mental health (Draper *op cit*.). The post‐war era also brought its own socio‐cultural ideas of emotion, including ‘keep calm and carry on’, having a ‘stiff upper lip’ and it not being socially acceptable to cry or show difficult feelings. For many older people growing up in this post‐war era, the strategy of keeping a lid on emotions and ‘just getting on’ may have worked for them throughout their lives, but this focus on ‘doing’ can become less accessible as older people navigate the ageing process.

Compassion, as a construct, has been shown to be correlated with the challenges to our emotion regulation system noted above, which are even more prevalent in later life. For example, in order to counteract loneliness, one needs not only social opportunities but also the ability to perceive both the other as a potential source of safeness and the self as deserving of connection and social soothing. The relevance of this multi‐directional flow of compassion has been demonstrated by Lee and colleagues; where higher baseline compassion‐to‐others (CTO) and compassion‐to‐self (CTS) as well as increases in CTO and CTS scores predicted lower loneliness scores at 5‐year follow‐up, as well as better mental well‐being (Lee et al., 2021).

Self‐compassion bears onto the inevitable physical health changes associated with older age. For people with poorer physical health and pain, self‐compassion is associated with greater subjective well‐being. Those with greater self‐compassion show greater willingness to use assistance for walking, hearing and memory and experienced greater life satisfaction and belief that they are ageing well (Allen et al., 2011). Allen's research also highlights the nuanced relationship between self‐compassion and disability; whereby the greater the level of suffering the greater the relevance of self‐compassion to adopting helpful behaviours. Towards the end of life and beyond, increasing self‐compassion has been shown to reduce death anxiety (Baharvandi et al., 2020), enable professionals to bear the emotionally and spiritually high‐demands of palliative care work (Buonaccorso et al., 2022) and reduce symptoms of complicated grief in the bereaved (Vara & Thimm, 2020).

## LITERATURE REVIEW

Given the relevance and apparent applicability of a compassion‐focused approach for working with older people in distress a review of the literature was conducted to explore the current state of the evidence‐base and to inform clinical and research recommendations going forwards.

### Literature search strategy

**FIGURE 1:**
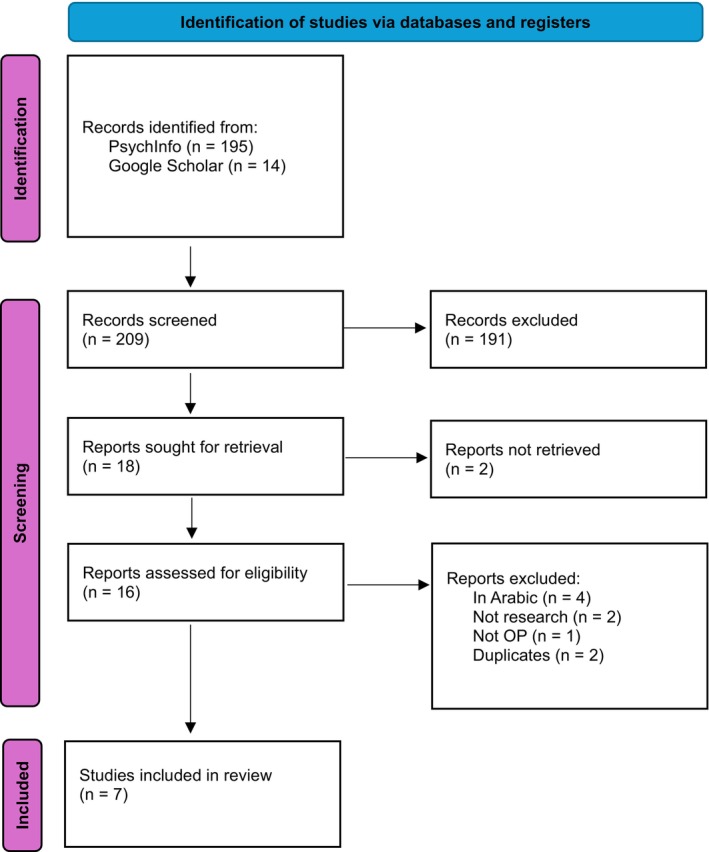
Prisma systematic review flow chart.

The terms ‘older people’, ‘older adults’, ‘elderly’ AND ‘compassion focused therapy’, ‘CFT’ were used on one database (PsychInfo), resulting in zero citations. The search term was widened by removing reference to older people, and only ‘compassion‐focused therapy’, ‘CFT’ were used, which resulted in 195 citations. Informal scoping was also conducted across Google Scholar (using terms ‘compassion focused therapy older people’ and ‘compassion focused therapy dementia’). All papers were screened for relevance (titles and abstracts) and were included if they evaluated CFT with older people. The following papers were excluded: studies investigating CFT with younger adults (under the age of 65), studies where the intervention was not CFT, papers not written in English, conceptual commentaries, book chapters and duplicates. Seven papers remained. See Figure 1 and Table 1.

**TABLE 1:** Literature review inclusion and exclusion criteria.

Inclusion criteria	Exclusion criteria
Intervention study using CFT	Other compassion interventions that are not CFT (i.e. Mindful Self‐Compassion, mindfulness etc.)
Participants are defined as OP	Participants not OP
*N* ≥ 1	Non‐intervention papers
Clinical population	

### Results

Table 2 summarises the findings of the electronic search. Of the seven papers identified, none were RCTs. There were two feasibility studies, two service evaluations and three case studies. There was a mix of group and individual CFT; three of the studies delivered CFT to OP with mental health difficulties in secondary care settings, three delivered the intervention to people with dementia within memory services and one was delivered to an individual with a functional motor disorder (in a secondary mental health care service). Of note, one paper evaluated CFT delivered in a lifespan service and so encompassed adults of working age and older people (Altavilla & Strudwick, 2022). The CFT interventions varied in duration, ranging from six weekly 2‐h sessions, to 28 1‐h sessions.

**TABLE 2:** Summary description of included studies.

Study	Design	*N*	Population	Treatment			Outcome measures	Main outcomes
				Intervention	Setting	Sessions		
Poz, R. (2018)	Case study	1	Early onset Dementia (with interpersonal difficulties and anger)	Individual CFT	Memory service	Unknown	Unknown	Less self‐to‐self and self‐to‐other criticism. Reduced avoidance
Collins, R., Gilligan, L. & Poz, R. (2017)	Service evaluation	64	Dementia (couples)	Group CFT	Memory service	6 weekly 2 h sessions	HADS, respiratory rate, QOL‐AD	Significant reduction in depression with a moderate effect 57% showed clinical improvement in anxiety and depression Reduction in respiratory rate with a large effect Quality of life improved with a large effect Positive effects in spouses also seen
Craig, C., Hiskey, S., Royan, L., Poz, P., Spector, A. (2018)	Feasibility case series	7	Dementia (with low mood and/or anxiety)	Individual CFT	Memory Services	10 weekly one hour sessions	CSDD, RAID, QOL‐AD, SCS‐SF, session rating form, interviews	Improvements in mood, anxiety and self‐compassion. No change in quality of life
Birdsey, N. (2020)	Case study	1	Older person with depression, anxiety, emotion‐regulation difficulties and physical health problems	Individual integrated CBT and CFT	Unknown	28 one hour sessions	PHQ‐9, GAD‐7	Depression and anxiety reduced from ‘severe’ but remained in clinical range. Greatest improvement occurred during CFT sessions
McConnell, F. (2021)	Feasibility study	13	Older people (60+) with anxiety and/or depression	Group CFT	OACMHT	10 90‐min weekly sessions	PHQ‐9, GAD‐7, FSCRS, SCS, Toronto Mindfulness Scale (TMS), Other as shame scale (OAS), Social Connectedness Scale revised	10/13 people completed the group, CFT a feasible and acceptable intervention to OA, 3/6 show reliable improvement in anxiety, no change in depression
Zarotti, N., Poz, R. & Fisher, P. (2022)	Case study	1	Older person (81) with motor functional neurological disorder	Individual CFT	Older People's Psychology Service	12 1 h sessions	Psychological Outcome Profiles, HADS, SCS	Reduction in perceived psychological impact of the mFND, clinically significant reduction in anxiety and depression, slight improvement in self‐compassion
Altavilla, A. & Strudwick, A. (2022)	Pilot group evaluation	23 (11 from later life services)	Lifespan (32–82 years old) with mental health difficulties (meeting secondary care criteria)	Group CFT	Secondary Care Mental Health Services	20 two and a half hour weekly sessions	SCS, Mindful Attention Awareness Scale, Depression and Anxiety Stress Scale, CORE‐34	Significant increase in the SCS and MAAS, significant decrease in depression subscale of the DASS and risk subscale of the CORE‐34. No significant differences in overall CORE and DASS scores

Two research trials were identified in the search, both of which were feasibility studies (Craig et al., 2018; McConnell, 2021). McConnell (2021) conducted a feasibility study in Scotland which was published as a DClinPsy thesis. The CFT intervention was delivered in a group format, across 10 90‐min weekly sessions. Thirteen participants were recruited from an older adult CMHT, with a variety of presenting difficulties, including depression, recurrent depression, generalised anxiety and mixed anxiety and depression. Participants were aged between 65 and 83 and nine of the 10 who agreed to participate in the research were female. Two separate groups were run, and they included core CFT principles and practices, including CFT psychoeducation (three systems model, noticing thoughts and feelings), CFT formulation, practices including mindful attention and soothing rhythm breathing, cultivating the compassionate self, multiple selves and exploring functions of self‐criticism. The groups were delivered by a principal clinical psychologist and a cognitive behavioural therapist experienced in CFT. Participants were given a workbook and audio practices to facilitate home practice. With regards to attendance, 77% attended six or more of the sessions, with only two attending all 10 sessions. Despite the study not being powered to detect change, the Reliable Change Index showed three of six participants saw a reliable improvement in anxiety. No significant improvements were seen in mood. Qualitative feedback included that the questionnaires were difficult/onerous to complete, but through attending the group participants developed a sense of safety and could give and receive compassion to and from other group members. The CFT group was positively received by all participants, but McConnell (2021) concluded there was insufficient evidence that this was related to core CFT components.

The second feasibility study was a case series of seven people with a diagnosis of dementia plus symptoms of depression and/or anxiety who were recruited across memory services in the South and East of England (Craig et al., 2018), which was also a DClinPsy thesis but published in a peer‐reviewed journal. CFT was delivered individually, across 10 weekly 1 h sessions. Most of the participants had a diagnosis of Alzheimers Disease (*N* = 5), one had vascular dementia and another had mixed dementia (vascular and Alzheimer type). The youngest participant was 58 years old with the oldest being 88; six of the seven participants were female. A CFT protocol was developed and adapted to dementia, for example, grounding the CFT psychoeducation in the context of dementia (such as an even ‘trickier brain’) and adapting its delivery to account for cognitive difficulties. Core CFT concepts were delivered, including three systems formulation, compassionate mind practices, developing compassion for the self and fears and blocks. The CFT was delivered by clinical psychologists and a trainee clinical psychologist all experienced in CFT and working therapeutically with people with dementia. Participants were given visual and audio resources to facilitate learning and home practice. Five participants completed all 10 sessions; however, none completed the full intervention in the allocated time. Six participants were able to engage in soothing rhythm breathing practices, and five were able to engage in discussion around self‐criticism and developing compassion. Of the six participants who had clinical symptoms of depression five saw a reduction in symptoms and three moved to the sub‐clinical range. All participants had clinical symptoms of anxiety and all saw a reduction in symptoms, with two moving to the sub‐clinical range. No changes were seen in quality of life. With regards to self‐compassion, this appeared to improve over the course of the intervention, with four participants seeing reliable change. Qualitative feedback indicated that overall the individual CFT intervention was experienced as positive, it being important that the clinician had knowledge of dementia, and experience of change in how they relate to themselves and to others. It was acknowledged that cognitive impairment may have made it more difficult for participants to process and engage with the material, as well as hold on to change. Some reported a wish for more psychological input upon the intervention ending.

A service evaluation with a large number of patients affected by dementia and their partners (*N* = 64) was published by Collins et al. (2017). They evaluated their CFT group run for couples affected by a dementia diagnosis in West Suffolk. The group ran for six weekly 2 h sessions, with the content covering core CFT components but being delivered flexibly depending on the need of the group. The groups were delivered by an experienced principal clinical psychologist and neuropsychologist (and an assistant psychologist or trainee clinical psychologist). Significant improvements were found across metrics, with over half showing clinical improvements in anxiety and depression and significant improvements in quality of life. Positive improvements were also found in the attending partners. The second service evaluation identified in the search was a pilot CFT group evaluation, delivered in a lifespan secondary care service, with 11 of the 23 patients coming from the later life service (Altavilla & Strudwick, 2022). This had the lengthiest treatment, with 20, two and a half hour weekly sessions. The groups were facilitated by two clinical psychologists who were experienced working within a CFT approach. Core CFT components were delivered, allowing time for practices, discussion, reflection and intention setting for home practices. The authors report significant improvements in self‐compassion and mindfulness as well as significant reduction in depression and expressions of suicidal feelings. However, these findings were for all ages and no separate analysis was conducted to see if improvements were found in the older adult population.

Three case studies were found in the search, one with a person with early‐onset dementia and interpersonal difficulties (Poz, 2018), another with an older person with depression, anxiety, emotion regulation difficulties and physical health problems (Birdsey, 2020) and another with an 81‐year‐old with a diagnosis of functional motor disorder (Zarotti et al., 2023). The number of sessions varied from 12 to 28 sessions and all report positive improvements for the individuals, including a reduction in self and other criticism, reduction in symptoms of depression and anxiety and reduction of perceived psychological impact of their condition. Whilst not a published peer‐reviewed study, early indications from two pilot groups for older people living with trauma and complex emotional needs are encouraging (Poz et al., in prep), mirroring the improvements that have been achieved in working‐age adult cohorts (e.g. Lucre, 2022; Lucre & Corten, 2013). Early analyses showed reduced DASS (Depression, Anxiety and Stress Scale) scores in all participants, along with shifts in Forms of Self‐Criticising/Attacking & Self‐Reassuring Scale (FSCRS) and suggestions that ‘hated self’ is the first to shift following a group CFT intervention (Poz et al., in prep). Of note there is also a funded NIHR trial underway on group CFT for people with dementia. We wait excitedly for the findings.

Despite the popularity of CFT as a therapeutic modality used across clinical (and non‐clinical) settings and its evidence of impact (e.g. Petrocchi et al., 2024), research on its effects in older people's settings is minimal. No RCTs have been published, with only two research studies and a small number of service evaluations and case studies. These are all important in beginning to demonstrate (or at least explore) a therapy's impact, and we commend the authors who have taken the time to publish their work. The papers summarised above indicate at least that CFT is a feasible and acceptable therapy for older people, both those with mental health difficulties and those with dementia. There is an indication that CFT (group and individual) may have positive effects on mental health symptoms and well‐being; however, more rigorous methodology is required before we can be more certain.

## CURRENT PRACTICE AND RECOMMENDATIONS

Whilst it is beyond the scope of this paper to instruct the reader in delivering CFT (see Gilbert & Simoris, 2022 for further guidance), we will speak to the adaptations that may be helpful with older people. Gilbert provides an overview of delivering CFT summarised into three core themes: (1) The therapeutic relationship (2) The formulation and practices of the therapy and (3) The goals of the therapy. This structure is followed below to map specific considerations for OP (see Table 3).

**TABLE 3:** Specific considerations for OP, with and without cognitive impairments, when delivering CFT.

Bonds and the therapeutic relationship	Tasks and practices	Goals and outcomes
Acknowledgement that the age of the therapist will almost always be less than the age of the patient, and some patients may find it counter‐intuitive to talk to a younger therapist. This needs to be worked through and therefore overtly named. The beauty of this age discrepancy is that it can bring more balance to the issue of power in the therapeutic relationship. Consider culture at both a macro level (e.g. ethnicity) and also a micro level (e.g. locality). Do your cultures align? Are you aware of any normative expectations of therapy? How does age intersect with these two factors? Identify what are the signals of ‘social grooming’ that would provide psychological safeness for this individual. More time may need to be given at the start or during sessions to make ‘small talk’. Attend to environmental issues that could disable and invite shame for an older person. For example, seats with arms to help with rising, space for wheelchairs to turn, clinic rooms with hearing loops, visually neutral flooring, adequate lighting for lip‐reading, accessible toilets. As a cohort, older people were required to attend fewer years of obligatory education and may have had less exposure to the ideas and jargon of therapy. This provides conditions which could invite shame. It is therefore more important to develop a secure therapeutic relationship before introducing psychoeducation elements or doing so more creatively.	This may be the first opportunity an older person has had to speak about decades‐long distress; with many patients finding this very difficult, yet helpful: More time needs to be anticipated before this can be gently approached. As people age, it is natural for speed of processing to slow, which can be even more pronounced in people with dementia. The process of therapy also needs to slow in order not to outpace the patient. However, this approach of slowing sits naturally with the process of CFT itself. Take time to explain therapeutic concepts and ensure they are returned to and/or repeated over the course of the therapy. Due to possible combinations of less familiarity with therapy concepts and increasing cognitive rigidity, it can be more important with older people to cover the basics of psychoeducation clearly and ‘show your workings’ when explaining how concepts connect and formulating collaboratively. Cognitive changes in normal ageing and more so in dementia, also affect the way in which older people can generate alternative possibilities. More of the effort therefore falls to the therapist to listen attentively and draw evidence from the narrative, that took place in a different emotional context to the current felt emotion. As language (expressive and receptive) is commonly affected in Alzheimer's dementia, consider additional ways to share psychoeducation and formulations, such as simple visual handouts and provide audio recordings of practices. In group formats using visual stimuli, such as pictures and slides can often trigger more ‘felt’ responses and trigger more memories than verbal conversations alone.	A core goal of CFT is to cultivate the three flows of compassion. Someone's (in)ability to identify with their future self can have long‐term consequences for their overall wellbeing (Reiff et al., 2020). The ability to turn towards the needs of 10 years hence self requires greater distress tolerance and self‐compassion for older people and those living with life‐limiting conditions, but is especially helpful to engage in advance decision‐ making. The goal of re‐balancing the emotion regulation system can feel tricky when an individual is facing multiple losses. Providing an understanding of the importance of joy, eudemonic and hedonic emotions, nurturing lived experiences of pleasure, playfulness and positive challenge, working with the person to identify sources of meaning and purpose, often advocating with them against a social discourse of ‘ageing gracefully and quietly in a corner’. Older people without cognitive impairments can usually access the standard CFT outcome measures, assessing factors such as self‐criticism and self‐reassurance, shame, fear of compassion (available on Compassionate Mind website). However, there is generally a preference to do fewer and shorter questionnaires, and some scaffolding may be needed with form completion. Older people with cognitive impairment have, in our experience, not been able to access these questionnaires, and outcome measures need to be significantly adapted to maximise on the individual's cognitive strengths; such as visual analogue scales or inviting qualitative feedback.
Sensory impairments may require the therapist to deliver the therapy in a counter‐intuitive way, for example, using a much louder voice when reflecting empathy and gentleness. Feedback from the CFT for dementia trial highlighted it was important that clinicians had a working understanding of dementia and its wide‐reaching effects on the individual and their system. Ensure that sufficient time is given to allow the person to discuss the meaning and impact of a dementia diagnosis	Although episodic memory capacity reduces somewhat with normal ageing and significantly with dementia, a strength of CFT for older people is that it draws on procedural memory, e.g. for breath work and on emotional memory systems, e.g. for compassionate other imagery, which can be explained to the patient remain more preserved. Even so, consider shorter practices, and remove long silences when working with people with dementia Breath work can hold even greater importance with older people. For example, there is a natural loss of height in spine (by an average of 3.5” in women), and the collapsing inwards and downwards of the shoulders into the chest cavity impacts on deep breathing. Therefore, two breathing practices per therapy session can be helpful. Emotional experiences can also be triggered though kinaesthetic memories, for example, introducing play‐doh to the session can fast‐track the person to the playfulness of their childhood, and stimulate the drive system. Compassionate postcards have been found to be an acceptable practice as part of individual and group CFT skills. The task of physically writing a postcard appearing to be conceptually acceptable to this cohort, but support may need to be provided for those with diminished fine‐motor skills, or who received less substantial formal education. Consider providing brief session summaries including any intersession tasks when working with those with cognitive impairment	Compassionate postcards have been shown to be appropriate for content analysis to provide insight into the experience of compassion. Irving‐Curran (2020) analysed the content of 75 postcards written in the final‐session of a CFT group for couples with dementia. This identified that participants experienced all three flows of compassion and identified their own active coping strategies. Consider inviting a ‘supportive other’ when working with a person affected by dementia. Not only can this build an understanding in the loved‐one but also they can also become an external memory‐aid, to facilitate practices such as noticing emotion states, as well as benefitting themselves from body and breath work and imagery practices

## SUMMARY AND CONCLUSIONS

The journey through the ageing process can pose challenges to navigate, including loss of roles, declining physical health and cognitive ability and loss of attachments. Older people may also have unprocessed early trauma and this period of reflecting on one's life may bring feelings of regret, disappointment and shame. CFT is well‐placed to facilitate older people to reflect on and explore their relationship to themselves, as well as how they care for others and allow others to care for them. This is increasingly important as the likelihood of developing a health condition which requires care increases with age and for those who are able to accept their limitations and relate to themselves with compassion, whilst also allowing themselves to be cared for by others, are likely to have a smoother journey and die well. The model of CFT also explains our evolutionary need throughout our entire lifespan for fun, joy and social playfulness, which can be eroded by functionalist societies. CFT gives ‘permission’ to attend to this need as part of therapy. Despite these clear indications for a CFT approach with older people, there is a dearth of quality research investigating its effects. This mirrors a general reluctance in the clinical psychology profession to work with OP, which can be considered within a CFT framework: the need to turn towards and sit with often painful issues of decline, loss and death, whilst maintaining a commitment to alleviate associated suffering.

The ageing of the global population is non‐negotiable. As a global community we need to develop the motivation to improve the lived experience for all its citizens across the lifespan including its oldest citizens. As a profession, we need to consider how we can cultivate this motivation amongst those entering the profession.

The published research studies show good evidence that CFT is a feasible modality for older people with and without cognitive impairment, with both evidence of statistical improvements in symptoms and importantly felt impacts for the people themselves. We recommend that clinicians publish their findings from CFT interventions to build sufficient evidence for funding for RCTs. A limitation of our review is that it did not follow PRISMA guidelines for systematic reviews (Page et al., 2021), instead it informally reviewed and summarised the literature. Once further studies have been published it would be pertinent to carry out a more formal systematic review of CFT for older people. To effectively deliver CFT to older people, some adaptations are recommended.

## AUTHOR CONTRIBUTIONS

**Rebecca Poz:** Writing – original draft; validation; writing – review and editing; conceptualization. **Catriona Craig:** Conceptualization; writing – original draft; methodology; validation; writing – review and editing; formal analysis; project administration.

## CONFLICT OF INTEREST STATEMENT

The authors declare no conflicts of interest.

## Data Availability

Data sharing is not applicable to this article as no datasets were generated or analysed during the current study.
